# A *P*2_1_2_1_2_1_ polymorph of (+)-clusianone

**DOI:** 10.1107/S1600536813031036

**Published:** 2013-11-23

**Authors:** Sree Vaneesa Nagalingam, Janet Wong Pik Ching, Mohammed Khaled bin Break, M. Ibrahim M. Tahir, Teng-Jin Khoo

**Affiliations:** a Center for Natural and Medicinal Product Research, School of Pharmacy, Faculty of Science, University of Nottingham Malaysia Campus, Jalan Broga, 43500 Semenyih, Selangor, Malaysia; b School of Chemical Sciences and Food Technology, Universiti Kebangsaan Malaysia, 43600 Bangi, Malaysia; c Department of Chemistry, Faculty of Science, Universiti Putra Malaysia, Malaysia

## Abstract

The title compound, C_33_H_42_O_4_ [systematic name: (1*S*,5*S*,7*R*)-3-benzoyl-4-hy­droxy-8,8-dimethyl-1,5,7-tris­(3-methyl­but-2-­enyl)bi­cyclo­[3.3.1]nona-3-ene-2,9-dione], has a central bi­cyclo­[3.3.1]nonane-2,4,9-trione surrounded by tetra­prenyl­ated and benzoyl groups. The compound was recrystallized several times in methanol using both a slow evaporation method and with a crystal-seeding technique. This subsequently produced diffraction-quality crystals which crystallize in the ortho­rhom­bic space group *P*2_1_2_1_2_1_, in contrast to a previous report of a structure determination in the *Pna*2_1_ space group [McCandlish *et al.* (1976 ▶). *Acta Cryst*. B**32**, 1793–1801]. The title compound has a melting point of 365–366 K, and a specific rotation [α]^20^ value of +51.94°. A strong intra­molecular O—H⋯O hydrogen bond is noted. In the crystal, mol­ecules are assembled in the *ab* plane by weak C—H⋯O inter­actions.

## Related literature

For related structural studies, see: McCandlish *et al.* (1976 ▶); Santos *et al.* (1998 ▶, 2001 ▶). For background to *Clusiacea*e metabolites, see: Monache *et al.* (1991 ▶); de Oliveira *et al.* (1996 ▶). For discussion of polycyclic polyprenylated acyl­phloroglucinols, including their biological properties, see: Piccinelli *et al.* (2005 ▶); Garnsey *et al.* (2011 ▶); Simpkins (2013 ▶).
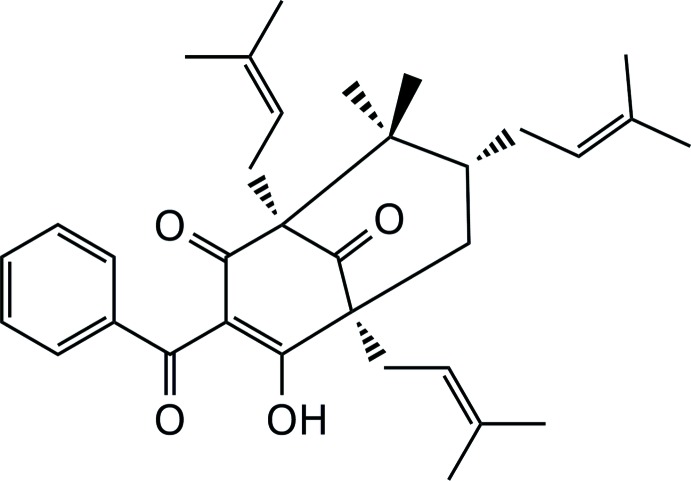


## Experimental

### 

#### Crystal data

C_33_H_42_O_4_
*M* *_r_* = 502.69
Orthorhombic, 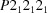

*a* = 9.2035 (2) Å
*b* = 13.4629 (2) Å
*c* = 22.9607 (5) Å
*V* = 2844.96 (10) Å^3^
*Z* = 4
Cu *K*α radiation
μ = 0.59 mm^−1^
*T* = 100 K
0.21 × 0.14 × 0.08 mm

#### Data collection

Oxford Diffraction Gemini diffractometer
Absorption correction: multi-scan (*CrysAlis PRO*; Oxford Diffraction, 2002 ▶) *T*_min_ = 0.86, *T*_max_ = 0.95
39826 measured reflections
5498 independent reflections
5269 reflections with *I* > 2.0σ(*I*)
*R*_int_ = 0.034

#### Refinement

*R*[*F* ^2^ > 2σ(*F* ^2^)] = 0.034
*wR*(*F* ^2^) = 0.084
*S* = 0.96
5498 reflections
335 parameters
H-atom parameters constrained
Δρ_max_ = 0.24 e Å^−3^
Δρ_min_ = −0.18 e Å^−3^
Absolute structure: Flack (1983 ▶), 2383 Friedel pairs
Absolute structure parameter: −0.04 (15)

### 

Data collection: *CrysAlis PRO* (Agilent, 2011 ▶); cell refinement: *CrysAlis PRO*; data reduction: *CrysAlis PRO*; program(s) used to solve structure: *SUPERFLIP* (Palatinus & Chapuis, 2007 ▶); program(s) used to refine structure: *CRYSTALS* (Betteridge *et al.*, 2003 ▶; Cooper *et al.*, 2010 ▶); molecular graphics: *CAMERON* (Watkin *et al.*, 1996 ▶) and *Mercury* (Macrae *et al.*, 2006 ▶); software used to prepare material for publication: *publCIF* (Westrip, 2010 ▶).

## Supplementary Material

Crystal structure: contains datablock(s) global, I. DOI: 10.1107/S1600536813031036/tk5270sup1.cif

Structure factors: contains datablock(s) I. DOI: 10.1107/S1600536813031036/tk5270Isup2.hkl

Supplementary material file. DOI: 10.1107/S1600536813031036/tk5270Isup3.cml

Additional supplementary materials:  crystallographic information; 3D view; checkCIF report

## Figures and Tables

**Table 1:** Hydrogen-bond geometry (Å, °)

*D*—H⋯*A*	*D*—H	H⋯*A*	*D*⋯*A*	*D*—H⋯*A*
O11—H1⋯O1	0.98	1.48	2.4227 (14)	158
C27—H273⋯O1^i^	0.99	2.63	3.330 (2)	128
C36—H363⋯O1^ii^	0.94	2.67	3.523 (2)	151
C8—H81⋯O11^iii^	0.95	2.62	3.300 (2)	129
C21—H211⋯O17^iv^	0.96	2.70	3.610 (2)	158

Symmetry codes: (i) 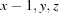; (ii) 
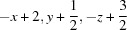
; (iii) 
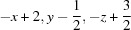
; (iv) 
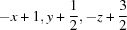
.

**Fig. 1.**
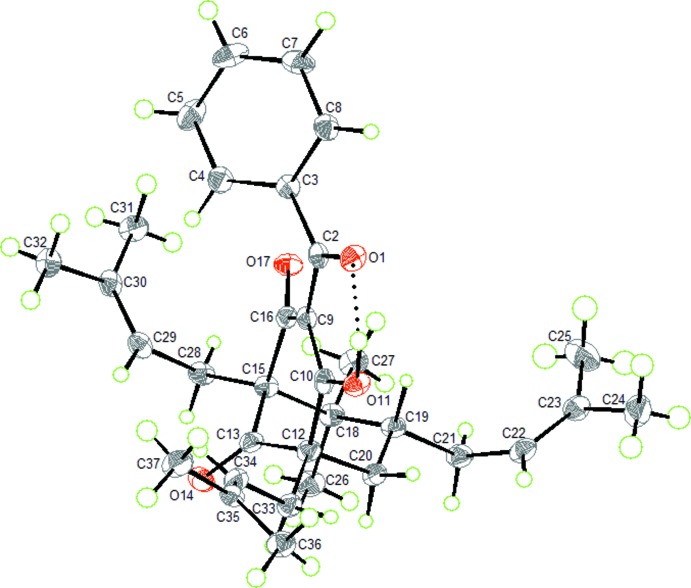
The title compound with displacement ellipsoids drawn at the 50% probability level. H atoms are shown as spheres of arbitrary radius. View of the molecule showing intramolecular hydrogen bond.

**Fig. 2.**
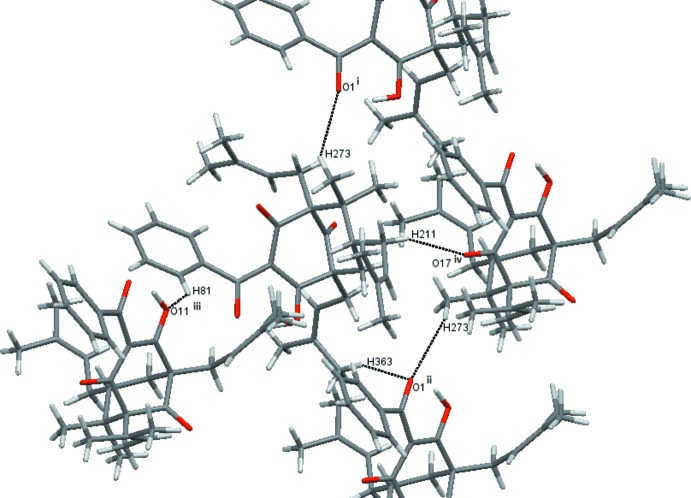
Partial packing diagram showing the C—H···O intermolecular interactions. Symmetry codes: (i) x - 1, y, z; (ii) -x + 2, y + 1/2, -z + 3/2; (iii) -x + 2, y - 1/2, -z + 3/2; (iv) -x + 1, y + 1/2, -z + 3/2.

## supplementary crystallographic information

### 1. Comment

Clusianone is a polycyclic polyprenylated acyl­pholroglucinols (PPAP) isolated from the plants of the family *Clusiacea*e (*Guttiferae*) and has gained considerable inter­est from both the natural product and synthetic chemistry community due to its potential bioactivity. Clusianone, both naturally occurring and synthetic, exhibits anti-HIV (Piccinelli *et al.*, 2005; Garnsey *et al.*, 2011) and anti-cancer properties (Simpkins, 2013).

Clusianone was isolated from the roots of *Clusia congestiflora* and analysis of X-ray diffraction has firmly established the equatorial orientation of the 3-methyl-2-butenyl group at C-7 which crystallized in the *Pna*2_1_ space group (McCandlish *et al.*, 1976). Subsequent isolation of the compound from *Clusia Sandiensis* (Monache *et al.*, 1991) and *Clusia spiritu-santensis* (de Oliveira *et al.*, 1996) led to NMR studies of clusianone and its methyl derivative, respectively, but gave contradictory NMR data for clusianone. Unfortunately, due to the complexity of the data and the unavailability of authentic sample of clusianone, no report was made of the C7 stereochemistry. Santos and co-workers isolated 7-epiclusianone from *Rheedia gardneriana* and reported its NMR and X-ray crystal structure, showing the C7 exo isomerism (Santos *et al.*, 1998; Santos *et al.*, 2001). Thereafter, the absolute structure of (+)-7- epiclusianone possessing an axial C7-prenyl group while comparison to clusianone isolated from the roots of *Clusia congestiflora* (McCandlish *et al.*, 1976) had the C7-prenyl group as equatorial. The title compound's epimer (+)-7- epiclusianone crystallized in space group of *P*2_1_2_1_2_1_ (Santos *et al.*, 1998).

To date, the absolute configuration of (+)-clusianone determined by X-ray crystallography at room temperature has been only confirmed by (McCandlish *et al.*, 1976). Previously reported clusianone isolated from *Clusia congestiflora* crystallizes in the *Pna*2_1_ space group. The crystal was obtained from a 95% ethanol solution (McCandlish *et al.*,1976). However, the lack of the specific optical rotation of the clusianone isolated by McCandlish *et al.* (1976) indicates that further investigation might be required to discover the uncertainty in stereochemistry of this compound.

Herein, we report the clusianone with melting point 365–366 K and a specific rotation [α]^20^ value of +51.94 °. We report (+)-clusianone to crystallize in the orthorhombic space group *P*2_1_2_1_2_1_, Fig. 1, when crystals were isolated from methanol solution. Intra­molecular O—H···O hydrogen bonds are noted, Table 1. Supra­molecular layers in the *ab* plane are stabilised by weak C—H···O inter­actions, Fig. 2. The different solvent used to crystallize the compound might be the reason for the polymorph occurance.

### 2.1. Isolation and crystallization

*G. Parvifolia* leaves were collected from trees in a reserved forest, Sungai Congkak, Selangor, Malaysia. The leaves (133 g) were dried, powdered and macerated with n-hexane (3 x 1.0 L) frequently over three days. Each maceration were filtered, evaporated and then dried using a rotary evaporator under reduced pressure at 40 °C. The hexane extract of the leaves (9.7 g) was then chromatographed on silica gel (70-230 mesh) and eluted with di­ethyl ether and evaporated. This fraction of the extract which contains a major portion of chloro­phyll compounds was then mixed with silica gel:activated charcoal in proportion of 1:3:1, respectively, and placed in a column with a porous frit. The material was eluted with hexane (500 ml) followed by di­chloro­methane (500 ml) with the aid of vacuum pressure. To isolate the compound, the di­chloro­methane dried fractions (4.8 g) was further chromatographed on silica gel (70-230 mesh) and eluted with mixtures of cyclo­hexane/chloro­form and chloro­form/methanol of increasing polarity. A total of 122 fractions were collected in 20 ml vials and fractions from F51—F60 were crystallized *via* slow methanol evaporation. Growth of diffraction quality crystals were obtained through several recrystallizations in methanol using both slow evaporation method and crystal seeding technique over a period of 10 days. Yellow cubic crystals (119 mg) were obtained and the melting point was 365-366 K and the specific optical rotation [α]^20^ was +51.94 °. The specific optical rotation was measured using an ADP-440 Perkin Elmer digital polarimeter using a sodium lamp at 589 nm. The melting point was recorded on Stuart's melting point apparatus SMP100. All the data analysis relevant to melting point, specific rotation and X-ray diffraction analysis were repeated three times to determine the reproducibility of the data and various parameters.

### 2.2. Refinement

Crystal data, data collection and structure refinement details are summarized in Table 1. The H atoms were all located in a difference map, but those attached to carbon atoms were repositioned geometrically. The H atoms were initially refined with soft restraints on the bond lengths and angles to regularize their geometry (C—H in the range 0.93–0.98 and O—H = 0.82 Å) and *U*_iso_(H) (in the range 1.2–1.5 times *U*_eq_ of the parent atom), after which the positions were refined with riding constraints except the hydroxyl hydrogen which were refined freely (Cooper *et al.*, 2010).

### Crystal data

**Table:**

C_33_H_42_O_4_	*D*_x_ = 1.174 Mg m^−^^3^
*M**_r_* = 502.69	Melting point: 365 K
Orthorhombic, *P*2_1_2_1_2_1_	Cu *K*α radiation, λ = 1.54180 Å
Hall symbol: P 2ac 2ab	Cell parameters from 19011 reflections
*a* = 9.2035 (2) Å	θ = 3–71°
*b* = 13.4629 (2) Å	µ = 0.59 mm^−^^1^
*c* = 22.9607 (5) Å	*T* = 100 K
*V* = 2844.96 (10) Å^3^	Prismatic, colourless
*Z* = 4	0.21 × 0.14 × 0.08 mm
*F*(000) = 1088	

### Data collection

**Table:**

Oxford Diffraction Gemini diffractometer	5498 independent reflections
Radiation source: Cu Ka	5269 reflections with *I* > 2.0σ(*I*)
Graphite monochromator	*R*_int_ = 0.034
ω scans	θ_max_ = 71.6°, θ_min_ = 3.8°
Absorption correction: multi-scan (*CrysAlis PRO*; Oxford Diffraction, 2002)	*h* = −11→11
*T*_min_ = 0.86, *T*_max_ = 0.95	*k* = −16→16
39826 measured reflections	*l* = −28→28

### Refinement

**Table:**

Refinement on *F*^2^	Hydrogen site location: difference Fourier map
Least-squares matrix: full	H-atom parameters constrained
*R*[*F*^2^ > 2σ(*F*^2^)] = 0.034	Method = Modified Sheldrick *w* = 1/[σ^2^(*F*^2^) + ( 0.04*P*)^2^ + 1.02*P*] , where *P* = (max(*F*_o_^2^,0) + 2*F*_c_^2^)/3
*wR*(*F*^2^) = 0.084	(Δ/σ)_max_ = 0.001
*S* = 0.96	Δρ_max_ = 0.24 e Å^−^^3^
5498 reflections	Δρ_min_ = −0.18 e Å^−^^3^
335 parameters	Absolute structure: Flack (1983), 2383 Friedel pairs
0 restraints	Absolute structure parameter: −0.04 (15)
Primary atom site location: other	

### Special details

**Table:**

Experimental. The crystal was placed in the cold stream of an Oxford Cryosystems open-flow nitrogen cryostat with a nominal stability of 0.1 K.

### Fractional atomic coordinates and isotropic or equivalent isotropic displacement parameters (Å^2^)

**Table:**

	*x*	*y*	*z*	*U*_iso_*/*U*_eq_	
O1	1.01156 (11)	0.23087 (8)	0.70014 (5)	0.0254	
H1	0.9976	0.3386	0.7123	0.0500*	
C2	0.89022 (15)	0.21427 (11)	0.67631 (6)	0.0196	
C3	0.87580 (15)	0.11723 (10)	0.64619 (6)	0.0196	
C4	0.81066 (17)	0.10926 (12)	0.59161 (7)	0.0252	
C5	0.81469 (18)	0.01993 (13)	0.56225 (7)	0.0333	
C6	0.87951 (19)	−0.06283 (13)	0.58714 (8)	0.0364	
C7	0.94348 (19)	−0.05514 (12)	0.64160 (8)	0.0338	
C8	0.94353 (17)	0.03472 (11)	0.67054 (7)	0.0260	
C9	0.78010 (15)	0.29113 (10)	0.67650 (6)	0.0189	
C10	0.82224 (15)	0.38637 (11)	0.69421 (6)	0.0200	
O11	0.95023 (11)	0.40387 (7)	0.71561 (4)	0.0244	
C12	0.72277 (16)	0.47533 (10)	0.69123 (6)	0.0207	
C13	0.60033 (16)	0.45383 (10)	0.64825 (6)	0.0204	
O14	0.56632 (12)	0.51181 (7)	0.61064 (5)	0.0285	
C15	0.51798 (15)	0.35827 (10)	0.66056 (6)	0.0198	
C16	0.62421 (15)	0.26982 (10)	0.66719 (6)	0.0187	
O17	0.57659 (11)	0.18581 (7)	0.66667 (5)	0.0232	
C18	0.43562 (16)	0.37607 (10)	0.72158 (6)	0.0215	
C19	0.55121 (16)	0.40042 (10)	0.76906 (6)	0.0211	
C20	0.64960 (16)	0.48756 (11)	0.75201 (6)	0.0220	
C21	0.48510 (17)	0.42104 (11)	0.83005 (7)	0.0255	
C22	0.59687 (18)	0.41885 (11)	0.87765 (7)	0.0268	
C23	0.61287 (19)	0.35035 (12)	0.91878 (7)	0.0297	
C24	0.7280 (2)	0.36145 (15)	0.96492 (8)	0.0415	
C25	0.5211 (2)	0.25871 (14)	0.92400 (9)	0.0454	
C26	0.32752 (17)	0.46277 (12)	0.71414 (7)	0.0286	
C27	0.35104 (16)	0.28311 (12)	0.73993 (7)	0.0275	
C28	0.41309 (16)	0.33433 (10)	0.60982 (6)	0.0227	
C29	0.48887 (16)	0.31514 (11)	0.55260 (7)	0.0234	
C30	0.48971 (16)	0.23037 (11)	0.52250 (6)	0.0235	
C31	0.41774 (19)	0.13577 (11)	0.54147 (7)	0.0297	
C32	0.56841 (18)	0.22262 (12)	0.46514 (7)	0.0286	
C33	0.80586 (17)	0.57064 (11)	0.67524 (6)	0.0238	
C34	0.90501 (18)	0.55841 (11)	0.62358 (6)	0.0269	
C35	1.03353 (17)	0.60074 (10)	0.61558 (6)	0.0242	
C36	1.10148 (18)	0.67277 (11)	0.65730 (7)	0.0283	
C37	1.1235 (2)	0.57708 (12)	0.56262 (7)	0.0336	
H41	0.7629	0.1648	0.5747	0.0322*	
H51	0.7720	0.0154	0.5245	0.0408*	
H61	0.8767	−0.1239	0.5662	0.0448*	
H71	0.9882	−0.1120	0.6603	0.0423*	
H81	0.9888	0.0410	0.7073	0.0323*	
H191	0.6141	0.3413	0.7739	0.0240*	
H201	0.7285	0.4939	0.7814	0.0255*	
H202	0.5948	0.5492	0.7515	0.0250*	
H211	0.4404	0.4855	0.8298	0.0302*	
H212	0.4098	0.3724	0.8376	0.0308*	
H221	0.6650	0.4724	0.8783	0.0333*	
H241	0.7808	0.4252	0.9604	0.0629*	
H242	0.6826	0.3587	1.0029	0.0630*	
H243	0.7968	0.3064	0.9624	0.0633*	
H251	0.4774	0.2571	0.9626	0.0712*	
H252	0.4405	0.2558	0.8975	0.0712*	
H253	0.5783	0.1996	0.9203	0.0711*	
H261	0.2489	0.4460	0.6869	0.0435*	
H262	0.3726	0.5228	0.7009	0.0443*	
H263	0.2793	0.4760	0.7516	0.0424*	
H271	0.2827	0.2989	0.7720	0.0415*	
H272	0.4133	0.2300	0.7529	0.0415*	
H273	0.2935	0.2588	0.7065	0.0413*	
H281	0.3488	0.3913	0.6046	0.0275*	
H282	0.3528	0.2790	0.6214	0.0274*	
H291	0.5415	0.3699	0.5369	0.0295*	
H311	0.3450	0.1161	0.5121	0.0463*	
H312	0.3641	0.1421	0.5785	0.0443*	
H313	0.4882	0.0812	0.5462	0.0471*	
H321	0.5028	0.1978	0.4355	0.0443*	
H322	0.6530	0.1781	0.4687	0.0436*	
H323	0.6061	0.2878	0.4517	0.0438*	
H331	0.8619	0.5902	0.7093	0.0268*	
H332	0.7355	0.6225	0.6683	0.0297*	
H341	0.8682	0.5150	0.5937	0.0329*	
H361	1.1371	0.7325	0.6379	0.0438*	
H362	1.1884	0.6436	0.6753	0.0454*	
H363	1.0391	0.6926	0.6877	0.0428*	
H371	1.1424	0.6355	0.5392	0.0501*	
H372	1.0793	0.5284	0.5373	0.0518*	
H373	1.2186	0.5520	0.5739	0.0518*	

### Atomic displacement parameters (Å^2^)

**Table:**

	*U*^11^	*U*^22^	*U*^33^	*U*^12^	*U*^13^	*U*^23^
O1	0.0188 (5)	0.0295 (5)	0.0279 (5)	0.0028 (4)	−0.0055 (4)	−0.0029 (4)
C2	0.0186 (7)	0.0247 (7)	0.0157 (6)	−0.0013 (6)	0.0009 (5)	0.0033 (5)
C3	0.0143 (6)	0.0228 (7)	0.0216 (7)	0.0013 (6)	0.0031 (5)	0.0010 (5)
C4	0.0223 (7)	0.0281 (8)	0.0250 (7)	0.0033 (6)	−0.0014 (6)	−0.0008 (6)
C5	0.0277 (8)	0.0415 (9)	0.0307 (8)	0.0014 (7)	−0.0041 (7)	−0.0124 (7)
C6	0.0330 (9)	0.0281 (8)	0.0480 (10)	0.0050 (7)	0.0038 (8)	−0.0156 (7)
C7	0.0331 (9)	0.0251 (7)	0.0432 (9)	0.0107 (7)	0.0017 (7)	0.0014 (7)
C8	0.0231 (7)	0.0299 (8)	0.0251 (7)	0.0045 (6)	−0.0002 (6)	0.0012 (6)
C9	0.0199 (7)	0.0209 (7)	0.0160 (6)	0.0006 (5)	0.0006 (5)	0.0015 (5)
C10	0.0192 (6)	0.0250 (7)	0.0157 (6)	−0.0026 (6)	0.0016 (5)	0.0013 (5)
O11	0.0215 (5)	0.0246 (5)	0.0270 (5)	−0.0021 (4)	−0.0028 (4)	−0.0040 (4)
C12	0.0222 (7)	0.0186 (7)	0.0215 (7)	−0.0026 (5)	0.0020 (6)	−0.0015 (5)
C13	0.0213 (7)	0.0186 (6)	0.0214 (7)	0.0034 (6)	0.0024 (6)	−0.0028 (5)
O14	0.0349 (6)	0.0215 (5)	0.0291 (6)	0.0015 (4)	−0.0056 (5)	0.0039 (4)
C15	0.0170 (6)	0.0196 (6)	0.0227 (7)	0.0005 (5)	−0.0002 (5)	−0.0006 (5)
C16	0.0194 (6)	0.0199 (6)	0.0169 (6)	0.0011 (5)	0.0005 (5)	−0.0007 (5)
O17	0.0195 (5)	0.0185 (5)	0.0318 (6)	−0.0007 (4)	0.0004 (4)	−0.0005 (4)
C18	0.0181 (7)	0.0214 (7)	0.0248 (7)	0.0010 (6)	0.0026 (6)	−0.0010 (5)
C19	0.0213 (7)	0.0179 (6)	0.0242 (7)	0.0021 (5)	0.0028 (6)	−0.0005 (5)
C20	0.0236 (7)	0.0200 (7)	0.0223 (7)	0.0000 (6)	0.0018 (6)	−0.0023 (5)
C21	0.0270 (7)	0.0222 (7)	0.0272 (7)	0.0026 (6)	0.0057 (6)	−0.0009 (6)
C22	0.0305 (8)	0.0241 (7)	0.0259 (7)	−0.0004 (6)	0.0081 (6)	−0.0059 (6)
C23	0.0340 (8)	0.0285 (8)	0.0267 (8)	0.0077 (7)	0.0078 (7)	−0.0029 (6)
C24	0.0491 (11)	0.0477 (10)	0.0278 (9)	0.0143 (9)	0.0009 (8)	−0.0037 (8)
C25	0.0507 (12)	0.0352 (10)	0.0503 (11)	0.0003 (9)	0.0050 (10)	0.0126 (8)
C26	0.0232 (7)	0.0291 (8)	0.0335 (8)	0.0066 (6)	0.0013 (7)	−0.0016 (7)
C27	0.0225 (8)	0.0293 (8)	0.0306 (8)	−0.0031 (6)	0.0043 (6)	−0.0013 (7)
C28	0.0178 (7)	0.0214 (6)	0.0290 (8)	0.0030 (5)	−0.0052 (6)	−0.0002 (6)
C29	0.0196 (7)	0.0252 (7)	0.0254 (7)	−0.0011 (6)	−0.0056 (6)	0.0039 (6)
C30	0.0187 (7)	0.0283 (7)	0.0235 (7)	0.0022 (6)	−0.0060 (6)	0.0012 (6)
C31	0.0312 (8)	0.0258 (7)	0.0322 (8)	0.0000 (7)	0.0025 (7)	−0.0028 (6)
C32	0.0283 (8)	0.0328 (8)	0.0247 (7)	−0.0003 (7)	−0.0015 (6)	0.0001 (6)
C33	0.0282 (8)	0.0199 (7)	0.0232 (7)	−0.0041 (6)	0.0019 (6)	−0.0015 (6)
C34	0.0381 (9)	0.0209 (6)	0.0217 (7)	−0.0045 (6)	0.0022 (7)	−0.0021 (6)
C35	0.0327 (8)	0.0173 (6)	0.0227 (7)	0.0020 (6)	0.0033 (6)	0.0028 (5)
C36	0.0280 (8)	0.0250 (7)	0.0317 (8)	−0.0024 (6)	0.0034 (6)	−0.0012 (6)
C37	0.0409 (9)	0.0267 (8)	0.0332 (9)	−0.0057 (7)	0.0124 (8)	−0.0030 (6)

### Geometric parameters (Å, º)

**Table:**

O1—C2	1.2635 (17)	C23—C24	1.506 (3)
H1—O11	0.984	C23—C25	1.500 (3)
C2—C3	1.4842 (19)	C24—H241	0.992
C2—C9	1.448 (2)	C24—H242	0.968
C3—C4	1.393 (2)	C24—H243	0.977
C3—C8	1.391 (2)	C25—H251	0.974
C4—C5	1.379 (2)	C25—H252	0.960
C4—H41	0.950	C25—H253	0.958
C5—C6	1.387 (3)	C26—H261	0.983
C5—H51	0.953	C26—H262	0.958
C6—C7	1.386 (3)	C26—H263	0.984
C6—H61	0.953	C27—H271	0.992
C7—C8	1.380 (2)	C27—H272	0.964
C7—H71	0.969	C27—H273	0.988
C8—H81	0.945	C28—C29	1.510 (2)
C9—C10	1.400 (2)	C28—H281	0.975
C9—C16	1.479 (2)	C28—H282	0.967
C10—O11	1.2979 (17)	C29—C30	1.334 (2)
C10—C12	1.509 (2)	C29—H291	0.953
C12—C13	1.526 (2)	C30—C31	1.500 (2)
C12—C20	1.5582 (19)	C30—C32	1.507 (2)
C12—C33	1.5381 (19)	C31—H311	0.987
C13—O14	1.2054 (18)	C31—H312	0.987
C13—C15	1.5198 (19)	C31—H313	0.986
C15—C16	1.5482 (19)	C32—H321	0.969
C15—C18	1.6110 (19)	C32—H322	0.986
C15—C28	1.5469 (19)	C32—H323	0.993
C16—O17	1.2131 (17)	C33—C34	1.506 (2)
C18—C19	1.558 (2)	C33—H331	0.973
C18—C26	1.543 (2)	C33—H332	0.966
C18—C27	1.533 (2)	C34—C35	1.326 (2)
C19—C20	1.5328 (19)	C34—H341	0.964
C19—C21	1.552 (2)	C35—C36	1.500 (2)
C19—H191	0.990	C35—C37	1.505 (2)
C20—H201	0.994	C36—H361	0.977
C20—H202	0.971	C36—H362	0.982
C21—C22	1.501 (2)	C36—H363	0.943
C21—H211	0.961	C37—H371	0.969
C21—H212	0.969	C37—H372	0.966
C22—C23	1.328 (2)	C37—H373	0.973
C22—H221	0.955		
			
O1—C2—C3	115.88 (13)	C22—C23—C25	124.43 (16)
O1—C2—C9	119.39 (13)	C24—C23—C25	114.94 (15)
C3—C2—C9	124.59 (12)	C23—C24—H241	110.9
C2—C3—C4	121.69 (13)	C23—C24—H242	109.1
C2—C3—C8	118.39 (13)	H241—C24—H242	109.8
C4—C3—C8	119.53 (14)	C23—C24—H243	109.8
C3—C4—C5	119.68 (15)	H241—C24—H243	109.4
C3—C4—H41	120.4	H242—C24—H243	107.7
C5—C4—H41	119.9	C23—C25—H251	108.9
C4—C5—C6	120.70 (15)	C23—C25—H252	114.7
C4—C5—H51	119.3	H251—C25—H252	104.9
C6—C5—H51	120.0	C23—C25—H253	111.5
C5—C6—C7	119.63 (15)	H251—C25—H253	106.8
C5—C6—H61	118.2	H252—C25—H253	109.5
C7—C6—H61	122.1	C18—C26—H261	111.8
C6—C7—C8	120.00 (15)	C18—C26—H262	113.2
C6—C7—H71	121.4	H261—C26—H262	108.1
C8—C7—H71	118.6	C18—C26—H263	109.3
C3—C8—C7	120.41 (14)	H261—C26—H263	105.5
C3—C8—H81	119.0	H262—C26—H263	108.7
C7—C8—H81	120.6	C18—C27—H271	110.6
C2—C9—C10	117.48 (13)	C18—C27—H272	112.9
C2—C9—C16	122.67 (13)	H271—C27—H272	107.8
C10—C9—C16	119.24 (13)	C18—C27—H273	109.2
C9—C10—O11	121.85 (13)	H271—C27—H273	108.0
C9—C10—C12	123.08 (13)	H272—C27—H273	108.3
O11—C10—C12	115.06 (12)	C15—C28—C29	113.75 (11)
H1—O11—C10	102.2	C15—C28—H281	107.9
C10—C12—C13	109.07 (11)	C29—C28—H281	107.9
C10—C12—C20	107.79 (11)	C15—C28—H282	108.1
C13—C12—C20	106.27 (11)	C29—C28—H282	111.9
C10—C12—C33	111.80 (12)	H281—C28—H282	107.0
C13—C12—C33	111.78 (12)	C28—C29—C30	126.86 (14)
C20—C12—C33	109.91 (11)	C28—C29—H291	115.6
C12—C13—O14	122.17 (13)	C30—C29—H291	117.5
C12—C13—C15	114.12 (11)	C29—C30—C31	124.98 (14)
O14—C13—C15	123.50 (13)	C29—C30—C32	120.98 (14)
C13—C15—C16	110.76 (11)	C31—C30—C32	114.03 (13)
C13—C15—C18	105.69 (11)	C30—C31—H311	109.2
C16—C15—C18	109.03 (11)	C30—C31—H312	113.4
C13—C15—C28	110.33 (11)	H311—C31—H312	105.8
C16—C15—C28	107.93 (11)	C30—C31—H313	112.0
C18—C15—C28	113.10 (11)	H311—C31—H313	108.7
C15—C16—C9	118.54 (12)	H312—C31—H313	107.4
C15—C16—O17	119.21 (13)	C30—C32—H321	109.7
C9—C16—O17	122.20 (13)	C30—C32—H322	110.4
C15—C18—C19	108.57 (11)	H321—C32—H322	110.0
C15—C18—C26	108.64 (12)	C30—C32—H323	112.3
C19—C18—C26	111.01 (12)	H321—C32—H323	107.7
C15—C18—C27	110.89 (12)	H322—C32—H323	106.7
C19—C18—C27	109.04 (12)	C12—C33—C34	113.47 (12)
C26—C18—C27	108.70 (12)	C12—C33—H331	107.3
C18—C19—C20	112.70 (11)	C34—C33—H331	110.0
C18—C19—C21	113.66 (12)	C12—C33—H332	108.0
C20—C19—C21	108.97 (11)	C34—C33—H332	110.8
C18—C19—H191	108.0	H331—C33—H332	107.0
C20—C19—H191	107.4	C33—C34—C35	127.09 (14)
C21—C19—H191	105.7	C33—C34—H341	114.5
C19—C20—C12	113.78 (11)	C35—C34—H341	118.4
C19—C20—H201	108.9	C34—C35—C36	124.15 (14)
C12—C20—H201	107.5	C34—C35—C37	120.78 (14)
C19—C20—H202	110.5	C36—C35—C37	115.06 (14)
C12—C20—H202	107.7	C35—C36—H361	112.4
H201—C20—H202	108.2	C35—C36—H362	110.5
C19—C21—C22	112.63 (12)	H361—C36—H362	104.3
C19—C21—H211	108.9	C35—C36—H363	113.8
C22—C21—H211	108.4	H361—C36—H363	108.0
C19—C21—H212	108.7	H362—C36—H363	107.3
C22—C21—H212	110.3	C35—C37—H371	112.1
H211—C21—H212	107.8	C35—C37—H372	113.5
C21—C22—C23	127.39 (15)	H371—C37—H372	106.9
C21—C22—H221	116.5	C35—C37—H373	110.7
C23—C22—H221	116.1	H371—C37—H373	105.5
C22—C23—C24	120.62 (16)	H372—C37—H373	107.6

### Hydrogen-bond geometry (Å, º)

**Table:**

*D*—H···*A*	*D*—H	H···*A*	*D*···*A*	*D*—H···*A*
O11—H1···O1	0.98	1.48	2.4227 (14)	158
C27—H273···O1^i^	0.99	2.63	3.330 (2)	128
C36—H363···O1^ii^	0.94	2.67	3.523 (2)	151
C8—H81···O11^iii^	0.95	2.62	3.300 (2)	129
C21—H211···O17^iv^	0.96	2.70	3.610 (2)	158

Symmetry codes: (i) *x*−1, *y*, *z*; (ii) −*x*+2, *y*+1/2, −*z*+3/2; (iii) −*x*+2, *y*−1/2, −*z*+3/2; (iv) −*x*+1, *y*+1/2, −*z*+3/2.

## References

[bb1] Agilent (2011). *CrysAlis PRO* Agilent Technologies UK Ltd, Yarnton, England.

[bb2] Betteridge, P. W., Carruthers, J. R., Cooper, R. I., Prout, K. & Watkin, D. J. (2003). *J. Appl. Cryst.* **36**, 1487.

[bb3] Cooper, R. I., Thompson, A. L. & Watkin, D. J. (2010). *J. Appl. Cryst.* **43**, 1100–1107.

[bb4] Flack, H. D. (1983). *Acta Cryst.* A**39**, 876–881.

[bb5] Garnsey, M. R., Matous, J. A., Kwiek, J. J. & Coltart, D. M. (2011). *Bioorg. Med. Chem. Lett.* **21**, 2406–2409. 10.1016/j.bmcl.2011.02.074 PMC3070777 21414776

[bb6] Macrae, C. F., Edgington, P. R., McCabe, P., Pidcock, E., Shields, G. P., Taylor, R., Towler, M. & van de Streek, J. (2006). *J. Appl. Cryst.* **39**, 453–457.

[bb7] McCandlish, L. E., Hanson, J. C. & Stout, G. H. (1976). *Acta Cryst.* B**32**, 1793–1801.

[bb8] Monache, F. D., Monache, G. D. & Gacs-Baitz, E. (1991). *Phytochemistry*, **30**, 2003–2005.

[bb9] Oliveira, C. M. A. de, Porto, A. M., Bittrich, V., Vencato, I. & Marsaioli, A. J. (1996). *Tetrahedron Lett.* **37**, 6427–6430.

[bb10] Oxford Diffraction (2002). *CrysAlis CCD* and *CrysAlis RED* Oxford Diffraction Ltd, Abingdon, Oxfordshire, England.

[bb11] Palatinus, L. & Chapuis, G. (2007). *J. Appl. Cryst.* **40**, 786–790.

[bb12] Piccinelli, A. L., Cuesta-Rubio, O., Chica, M. B., Mahmood, N., Pagano, B., Pavone, M., Barone, V. & Rastrelli, L. (2005). *Tetrahedron*, **61**, 8206–8211.

[bb13] Santos, M. H., Nagem, T. J., Braz-Filho, R., Lula, I. S. & Speziali, N. L. (2001). *Magn. Reson. Chem.* **39**, 155–159.

[bb14] Santos, M. H., Speziali, N. L., Nagem, T. J. & Oliveira, T. T. (1998). *Acta Cryst.* C**54**, 1990–1992.

[bb15] Simpkins, N. S. (2013). *Chem. Commun.* **49**, 1042–1051. 10.1039/c2cc37914g 23229029

[bb16] Watkin, D. J., Prout, C. K. & Pearce, L. J. (1996). *CAMERON* Chemical Crystallography Laboratory, Oxford, England.

[bb17] Westrip, S. P. (2010). *J. Appl. Cryst.* **43**, 920–925.

